# Can Public–Private Partnership Wastewater Treatment Projects Help Reduce Urban Sewage Disposal? Empirical Evidence from 267 Cities in China

**DOI:** 10.3390/ijerph19127298

**Published:** 2022-06-14

**Authors:** Xinshuo Hou

**Affiliations:** Business School, Xiangtan University, Xiangtan 411105, China; hxs@xtu.edu.cn

**Keywords:** treatment effect, moderating mechanism, project demonstration, fiscal pressure, operational efficiency

## Abstract

Human activities have placed enormous pressure on the world’s water resources. To improve the efficiency of water supply and wastewater treatment, public–private partnerships (PPPs) are widely used for sewage treatment. However, an academic question remains about whether PPP sewage treatment projects (PPPSTs) help reduce urban sewage disposal when responsibilities shift from the public sector to the private sector. This study used panel data of 267 prefecture-level cities in China from 2009 to 2020 to construct a difference-in-difference (DID) model based on the counterfactual framework to answer this question empirically. The model results significantly support the effect of PPPSTs on sewage disposal reduction. Furthermore, these results passed the parallel trend test and the placebo test, and the results were still achieved when the quadratic term of the core variable was introduced, indicating that the model is reliable. In addition, the moderating effect models were used to expand the analysis. That is, the regressions were derived by multiplying the relevant extended variables and the core independent variables. This analysis indicates that the operation mode of PPPST and the characteristics of national demonstration play an essential role in reducing the amount of urban sewage disposal. However, the effect of fiscal decentralization is not apparent. These conclusions were also confirmed in the model using the investment scale of PPPSTs. Therefore, paying attention to the formation of PPPST contracts and adopting a practical supervision system is of great significance for improving the effect of sewage disposal reduction.

## 1. Introduction

Urban sprawl leads to an increase in sewage disposal volume [1] and demand for sewage treatment facilities [2,3], making it an essential part of the construction of urban ecological civilization [4]. Human health, human security, economic development, and the development of tourism and recreation are all closely related to the appropriate quantity and quality of water resources [5,6]. Countries have made efforts in terms of many related aspects. One category of literature focuses on technical means, such as improving the treatment process of sewage treatment plants, which is a crucial way to improve the efficiency of sewage treatment from scientific and technological perspectives [7]. Li et al. [8] examined the recent development and prospects of drainage services departments by evaluating how different forms of renewable energy can be harnessed. Su et al. [9] studied the application of solar energy in sewage treatment plants. Bressani-Ribeiro et al. [10] developed a sustainable wastewater treatment process to address Brazil’s massive shortage of sanitation infrastructure. Sarpong et al. [11] proposed a wastewater treatment plant with the potential for energy recovery from a small wastewater treatment system. Another category of literature, about different approaches to urban sustainability and wastewater management, explores implementations in various countries to improve the efficiency of water supply and wastewater treatment. Relevant studies note that it is necessary to improve the technology, management, operation, and monitoring mechanism of sewage treatment to ensure that sewage disposal meets the prescribed standards [12]. In Poland, for example, a guide to the activities of companies in the water and wastewater sector can be found, and it is emphasized that legislation for the health of present and future generations requires the control of the return of treated water to the natural environment [5]. In China, a water resource management strategy called Sponge City has been implemented [13]. It is inspired by low-impact development, green infrastructure, sustainable drainage systems, and water-sensitive urban design. In Rio de Janeiro, Brazil, sewage treatment is being undertaken by providing universal access to private concessions for clean water, sanitation, and health [14].

From the above literature, proper management of water utilities or openness to innovation may also be a factor in improving efficiency. Companies that supply water and treat wastewater operate in a specific environment, which is a monopoly. While they have a legal obligation to provide water and treat wastewater, they should not lose money from an economic point of view [6]. Depending on the country and region, different legal tools have been introduced to regulate the operation of such companies to varying degrees [5]. Among them, the PPP model is widely used in the management process of water pollution treatment projects.

In response to government fiscal pressures and to improve industrial efficiency, an increasing number of public–private partnerships (PPPs) are being used for wastewater treatment [15,16]. The public sector can save money and technology through PPPs to achieve sustainable development [17]. In contrast, the private sector can reap long-term returns and enhance the reputation of companies through PPPs [18]. Sewage treatment is one of China’s earliest public service fields to apply PPPs. In addition, the Chinese government has issued policy documents, such as the “Implementation Opinions on Promoting Government-Private Partnerships in the Field of Water Pollution Prevention and Control” and the “Water Pollution Prevention and Control Action Plan”, to encourage public–private partnership sewage treatment projects (PPPSTs) in the field of water pollution prevention and control, leading to the large-scale development of PPP projects in China in terms of public service provision, especially after 2014. Accordingly, as of the end of 2020, a total of 1294 sewage treatment-related projects were retrieved from China’s PPP management database, of which, 1215 projects were initiated and implemented in 2015 or later. The development of PPP projects in the field of sewage treatment is inseparable from the government’s encouragement of social capital to invest in water environmental protection, and PPPs have become one of the fastest-growing public service delivery mechanisms in sewage treatment.

PPP projects in the water sector have received increasing attention in the literature and have been used in developing countries [19,20]. Scholars are more concerned about the discussion of PPP projects to improve the quality and efficiency of public services [19,21] and believe that the participation of the private sector will help to solve the problem of low quality of service supply caused by the government’s monopoly in public service supply [22]. Some scholars have expressed supportive attitudes and believe that the advantages of the PPP model mainly lie in (a) the introduction of a market competition mechanism to stimulate allocation efficiency [23], (b) improving the inefficient management of the public sector [24], and (c) stimulating the occurrence of innovation [25]. For example, Tang et al. used the enterprise-level data of sewage treatment plants in Jiangsu Province from 2011 to 2015 to examine the service quality of sewage treatment under PPPs. They believed that there was a positive effect [1]. The study of Tang et al. is the most relevant research to this paper, but this paper differs significantly from their work in terms of research subjects and research methods. Scholars who hold different opinions believe that PPPs may result in additional uncertainties, such as transaction costs, financing costs, and risk costs [26,27]. Because private sector participation in PPP projects is lucrative, it is easy for the private sector to focus too much on economic goals and adversely affect the environment [28]. In addition, the unfavorable conditions of PPP projects, such as renegotiation issues, cost overruns, and demand risks, have also been theoretically discussed [29]. The objective function that attaches importance to private interests makes scholars doubt the service quality assurance of private suppliers in PPP projects [30,31]. However, few studies have been undertaken on the effects of PPP projects on water pollution and water environment governance [28].

When the responsibilities of the public sector are shifted to the private sector in PPP programs, the public interest becomes more fragile and sensitive [32,33]. So, will the introduction of PPPSTs help reduce urban sewage disposal? This study used urban sewage disposal as the dependent variable to answer this question. It used a quasi-natural experiment based on cities that have launched PPPST projects to construct a difference-in-difference model (DID), and empirically evaluated whether the PPP model benefits urban sewage emission reduction using the economic and sewage disposal data of prefecture-level cities in China from 2009 to 2020. The study found that the participation of the private sector has a high probability of reducing urban sewage disposal. The treatment effect value indicates that, after controlling for the relevant influencing factors, such as economic development level, population size, and industrial structure, urban sewage disposal can be statistically reduced by 3.6%. In further discussion, the moderating effects of the operating mode of PPPSTs, the degree of fiscal decentralization, and the presence of the national-level demonstration PPPSTs are identified and discussed. The analysis indicates that the moderating effect of the operating mode on urban sewage disposal is significantly negative, suggesting that different operation modes may lead to different incentives for private suppliers. The fiscal factor reflected in the degree of urban fiscal decentralization does not show a sufficiently significant heterogeneous impact. Although alleviating financial pressure is an essential reason for cities to apply PPPSTs, the financial situation itself does not appear to be related to the effect of PPPSTs on wastewater abatement. National-level demonstration projects play a significant role in the sewage reduction effect of PPPSTs, which may be related to the demonstration nature of those projects.

Therefore, this study proposes the following hypotheses:

**Hypothesis** **1.** *PPPSTs can enhance the reduction in urban sewage discharge.*

**Hypothesis** **2.** *The wastewater emission reduction effects of PPPSTs may vary according to city-related characteristics, such as project operation mode, city financial situation, and whether there are national demonstration projects.*

The main differences between this paper and related research, and its innovative contributions, are in terms of three aspects. First, by examining the information on PPP projects in the field of sewage disposal provided by the China PPP Center, this study provides more detailed and specific econometrical evidence about whether PPPs have had a real impact on reducing sewage treatment, as opposed to existing research methods that focus on statistical descriptions. Second, the advantage of using nationwide city-level data of China for the empirical analysis is that potential heterogeneity among provincial cities can be controlled better than when using national or provincial macro data. Given the wide variation in levels of development and resource endowment between cities, this approach allows for a more accurate analysis of the fundamental role of PPPSTs, a larger sample size, and greater generality of the research than is available in current studies. Third, the DID model based on the counterfactual framework, which is a reliable causal identification method used in economic analysis, can help identify the impact of the implementation of PPPSTs on urban sewage disposal. When considering the cumulative amount of PPST investment per city, the heterogeneity of disposal reduction effects can be further identified from the scale of the investment. In addition, the detailed information on urban PPPST projects can also be used to discuss the moderating effects of different operation modes, urban fiscal decentralization, and national-level demonstration projects.

The remainder of this article is arranged as follows. Section 2 describes the development status of China’s sewage treatment PPP projects; Section 3 presents the construction of the empirical analysis model and introduces the variables and data. Section 4 presents the basic analysis results and related tests. Section 5 expands the research. Then, the discussion is presented in Section 6. Finally, Section 7 provides the conclusions and limitations of this study.

## 2. Evolution of PPPSTs in China

Sewage treatment has always been an issue of great concern to governments in the process of urban planning and construction. However, especially due to the rapid expansion of cities in China, the pressure resulting from the pollution of the urban water environment has increased significantly because China’s water resources are relatively tight, and the problem of water pollution is a serious one under urbanization and industrialization processes. In the past, local governments in China took full responsibility for the investment and management of municipal infrastructure, which exerted tremendous pressure on the governments’ fiscal positions. At the same time, it was not easy to keep up with management [34]. As a result, urban water pollution has become an important factor restricting the sustainable development of China’s economy and society.

**Figure 1:**
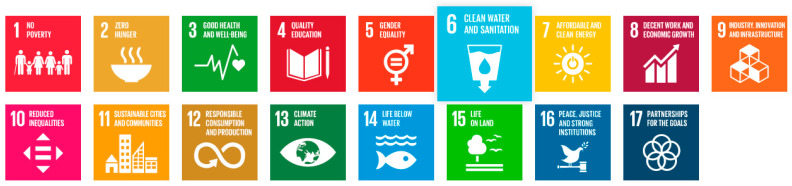
The Sustainable Development Goals. Note: The figure was accessed from https://www.genevaenvironmentnetwork.org/environment-geneva/key-areas-sdg/ (accessed on 17 March 2022).

Furthermore, ensuring the availability and sustainable management of water and sanitation services for all has become one of the Sustainable Development Goals, i.e., SDG6 [35]; see Figure 1. Empirical evidence in many countries has proven that urban water supply facilities are often poorly managed and inefficient [36]. In continuous practice, the introduction of PPPs into sewage treatment has become a key solution to compensate for the huge capital investment problem in recent years [37].

To relieve the increasing pressure on the government to provide infrastructure for water pollution treatment, PPP projects have been scaled up in the provision of public services, especially in China since 2014. On 23 September 2014, the Ministry of Finance of the People’s republic Of China issued the “Notice on Issues Concerning the Promotion and Application of the Cooperation Model of Government and Social Capital” (Caijin [2014] No. 76). The document pointed out that “social investors are encouraged to participate in the investment and operation of public welfare undertakings with certain benefits such as urban infrastructure through franchising and other means” and requires fiscal departments at all administrative levels to focus on urban sewage treatment and other infrastructure and public service areas, and to promote the use of government–social capital cooperation modes. Readers can obtain more detailed information at http://jrs.mof.gov.cn/zhuanti2019/ppp/zcfbppp/201410/t20141031_1155346.htm (accessed on 17 March 2022)

On 9 April 2015, the Ministry of Finance of the People’s republic Of China issued the “Implementation Opinions on Promoting Government-Private Partnership in the Field of Water Pollution Prevention and Control” (Caijian [2015] No. 90) to encourage the promotion of PPPs in the field of water pollution prevention and control, as detailed in http://jrs.mof.gov.cn/zhuanti2019/ppp/zcfbppp/201504/t20150428_1224072.htm (accessed on 17 March 2022).

On 16 April 2015, the “Water Pollution Prevention and Control Action Plan”, issued by the State Council of the Chinese government, further pointed out that, in the prevention and control of water pollution, it is necessary to promote diversified financing and guide social capital to increase investment in water environmental protection. The document is available at http://www.gov.cn/zhengce/content/2015-04/16/content_9613.htm (accessed on 17 March 2022).

In addition, on 19 July 2017, the Ministry of Finance of the People’s republic Of China issued the “Notice on the Comprehensive Implementation of the PPP Model for Sewage and Waste Treatment Projects Involved by the Government”, which can be accessed through http://www.gov.cn/xinwen/2017-07/19/content_5211736.htm (accessed on 17 March 2022). This notice indicated that the market mechanism should be introduced into sewage treatment in an overall manner to fully attract the participation of social capital. Its purpose is to standardize the market operation of the sewage treatment industry and improve the efficiency of government participation. Therefore, it is planned to fully implement the PPP mode for the governments’ sewage treatment projects, form an efficient PPP market dominated by social capital, and promote a significant optimization of the supply structure of related environmental public products and services.

**Figure 2:**
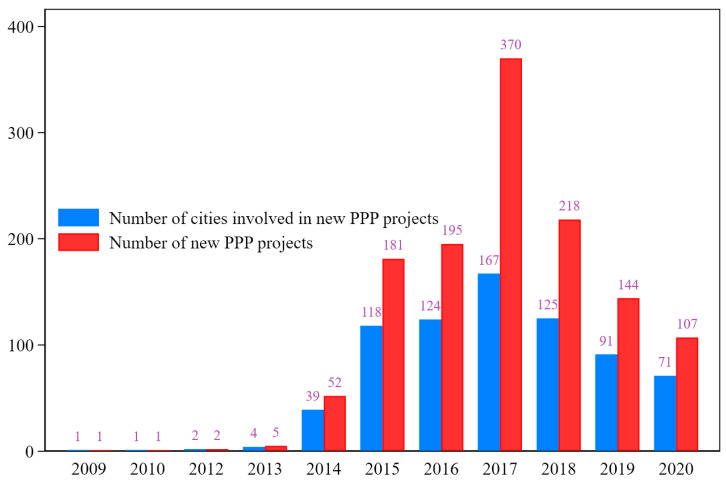
Annual distribution of PPPSTs in China. Note: The data is compiled from the PPP project management database of the Ministry of Finance of the People’s Republic of China (accessed on 8 September 2021); the author drew the figure via STATA 16.0 (StataCorp LLC, College Station, TX, USA).

Based on the implementation of the above policy documents, Chinese urban governments have vigorously promoted the comprehensive management of the water environment in recent years [34]. Figure 2 shows the distribution of the number of PPPSTs in China each year. From the perspective of the number of projects, only a minimal number of PPPSTs were implemented before 2014, i.e., a total of nine projects; there was a sharp increase after 2014, especially in 2017. From the perspective of the number of cities, PPPSTs have gradually been promoted in many cities, and the number of cities and the number of projects show a consistent trend. Clearly, the cities involved are repeated in different years; that is, some cities have multiple PPPSTs. According to the statistics, by the end of 2020, a total of 233 cities in China had initiated PPPSTs.

**Figure 3:**
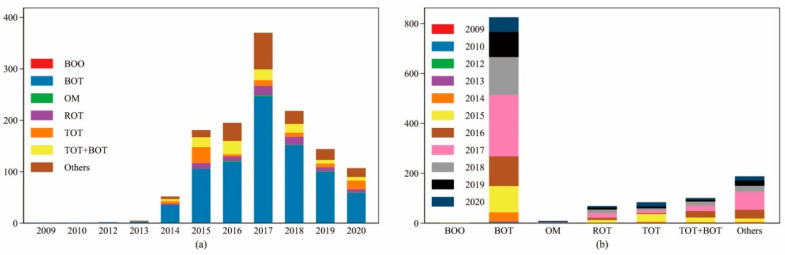
Distribution of PPPSTs in China: (**a**) sorted by year, (**b**) sorted by type. Note: The author drew the figure via STATA 16.0 (StataCorp LLC, College Station, TX, USA).

From the perspective of the operating mode, PPPSTs mainly include BOO, BOT, OM, ROT, TOT, TOT + BOT, and others. Table 1 explains the basic meaning of each type, and Figure 3 shows their distribution, sorted by year and by type of operation. It can be found that China’s PPPSTs are mainly based on the BOT operating mode, followed by TOT + BOT, TOT, ROT, etc. Each year, BOT has also been selected as an essential operation mode. According to the statistics, PPPSTs operated by BOT account for 64.65% of the sample in this study, and the three categories of TOT + BOT, TOT, and ROT account for a total of 19.14%, which means that, in China, at least 83.79% of PPPSTs have the operation mode of eventually transferring ownership to the government, which is also one of the characteristics of China’s PPPSTs. Therefore, in this study, it is believed that it may be interesting to examine whether different operation modes affect the impact of PPPSTs on urban sewage disposal; this is tested later.

**Figure 4:**
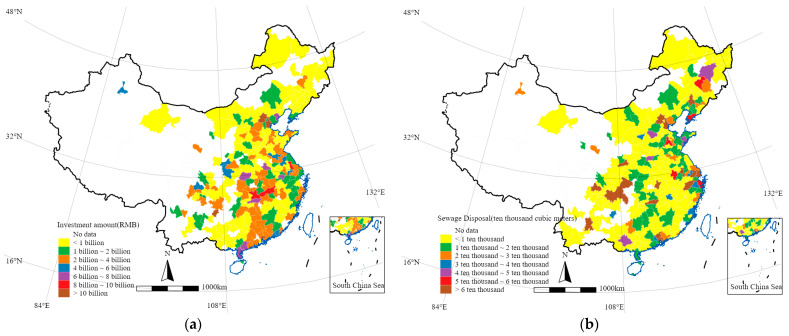
Spatial distribution of (**a**) investment amount of PPPSTs, and (**b**) urban sewage disposal. Note: The author drew figures via STATA 16.0 (StataCorp LLC, College Station, TX, USA).

Figure 4 shows the spatial distribution of total investment in PPPSTs in Chinese cities by the end of 2020 (Figure 4a) and the spatial distribution of sewage disposal (Figure 4b), and also presents the empirical sample distribution used in this study. It can be found that the distribution of PPPST investment has noticeable geographical differences in space, and this is also true for the distribution of urban sewage disposal. Furthermore, the distribution rules of the two are not entirely corresponding, so it is necessary to explore the relationship between the two variables through regression analysis.

In China, unbalanced regional development is an important problem in the sewage treatment industry. Among the regions, the economically developed regions, such as the eastern coastal areas, have solid fiscal strengths and dense populations suitable for the construction and operation of large-scale sewage facilities. In contrast, the central and western regions are relatively underdeveloped, with limited fiscal strength, scattered populations, and underdeveloped sewage treatment facilities. Figure 4a shows the spatial layout of the amount of investment in PPPSTs in China at the city level. It can be seen that the distribution is relatively extensive, and there is also a distribution of relatively high investment amounts in some central and western regions, indicating that, despite the pressure and restrictions on the level of economic development, actively adopting PPPs may effectively make up for the lack of public service investment. Figure 4b shows the spatial distribution of sewage disposal. Cities with high sewage disposal are mainly distributed in the eastern coastal areas, and there is also some scattered distribution in the central and western regions. Therefore, in the context of cities facing different pressures on sewage treatment, adopting PPPSTs may be more meaningful, especially for cities in the underdeveloped regions of the central and western regions.

## 3. Materials and Methods

### 3.1. Estimation Methods

This study views urban PPPSTs as a public policy intervention, and a quasi-natural experiment that fits within the framework of counterfactual analysis. Therefore, the difference-in-difference (DID) method, which is widely used in the evaluation of policy effects, can be used to evaluate the impact of PPPSTs on urban sewage disposal. DID has been widely used [38,39], and its principle is to construct a treatment group with intervention, and a control group without intervention. In this setting, the model reveals policy effects by controlling for other factors and comparing the differences between the treatment and control groups before and after the policy.

In many cases, there are differences in the time when the research objects are implemented, and the policy is gradually promoted, which constitutes a staggered DID model. For example, Kudamatsu [40] took advantage of the gradual push of the democratic wave when studying the impact of democratization on infant mortality, thereby addressing the endogenous problem of democratization affecting economic growth and social development. This method is a crucial means of identifying causal relationships within the framework of counterfactual analysis.

In this study, it was necessary to know the outcome, treatment, and post elements. The outcome is the dependent variable, reflecting the evaluated index. The treatment is used to distinguish treatment groups from control groups, and the post element is used to mark whether it is the period after policy intervention occurs. When assessing the effect of the treatment on the outcome, the control group is a potential counterfactual baseline frame of reference. At this point, the DID model can be easily constructed. Specific to the design of this study, the years in which PPPSTs were launched for cities are different, meaning the treatment is binary and varies over time, thus forming a multi-period staggered DID. In this study, the logarithmic indicator of urban sewage disposal (*LNSW*) was used as the dependent variable and a binary dummy variable (*WPPP*) proxy was used for the core independent variable. Here, *WPPP_it_* reflects whether city *i* initiated PPP wastewater treatment projects in year *t*: the value is 1 in the year of initiation, and otherwise 0 in subsequent years. Although it is difficult to provide a unified and clear post variable setting to the control group under the staggering situation, the model is usually estimated under two-way fixed effects. The essence of *WPPP* in this situation is the indicator variable, which, when the treatment group samples received the intervention, is the multiplication term corresponding to *treat* × *post*. This setting automatically produces the DID term between the treatment group and the control group, and before and after initiation. Now, the estimation model can be briefly described as Function (1), referring to the study of Angrist and Pischke [41].

(1) $$Y_{it}=\alpha +\delta \times treat_{it}\times post_{it}+c_{i}+\lambda_{t}+\varepsilon_{it}\overset{}{\to }Y_{it}=\alpha +\delta \times WPPP_{it}+c_{i}+\lambda_{t}+\varepsilon_{it}$$

where *Y* is the outcome variable, *treat* is the dummy variable that distinguishes the treatment group from the control group, and *post* is the dummy variable that distinguishes the period before and after the intervention occurs; the subscript *i* represents the city, *t* represents the year; *c_i_* and *λ_t_* represent the city and year fixed effects, respectively; *ε* is the random disturbance term. The coefficient δ reflects the treatment effect level established by PPPSTs on city sewage disposal. Function (2) shows the calculation principle of the average treatment effect (ATE) expressed as a coefficient [41,42]; that is, it reflects the difference in sewage disposal between the treatment group and the control group before and after the city initiated PPPSTs. Here, the subscript of $Y_{1}^{T}$ indicates that the sample belongs to the treatment group (*treat* = 1), and its superscript indicates that the sample is in the treated state (*post* = 1); the same manner is used to understand several other terms.

(2) $$\begin{array}{l} \delta =\{E[Y_{it}|treat=1,post=1]-E[Y_{it}|treat=1,post=0]\}\\ -\{E[Y_{it}|treat=0,post=1]-E[Y_{it}|treat=0,post=0]\}\\ =(Y_{1}^{T}-Y_{0}^{T})-(Y_{1}^{C}-Y_{0}^{C})=(\delta +\lambda_{t})-\lambda_{t}\\ =(Y_{1}^{T}-Y_{1}^{C})-(Y_{0}^{T}-Y_{0}^{C})=(\delta +c_{i})-c_{i} \end{array}$$

When the core independent variable is replaced by continuous variables, such as the investment amount of PPPSTs, this model can constitute the basic form of continuous DID. Qian et al. [43] pointed out that replacing binary dummy variables with continuous variables has all the advantages of the traditional DID model. At the same time, it can be used to consider the heterogonous effects caused by the differences in the degree of intervention. In this scenario, the key point is that the city does not only consider whether to initiate PPPSTs, but further considers the issue of the scale of investment. Then, the impact of different PPPST investment scales on urban sewage disposal can be further analyzed.

### 3.2. Model and Variables

The IPAT model, which can be expressed as Impact = Population × Affluence × Technology, is widely regarded as the basic framework for studying the impact of economic activities on the environment [44,45], and the model is modified to consider stochastic impacts by regression on population, affluence, and technology; that is, the STIRPAT model. It can be shown as Function (3), with *I* representing the outcome variable of concern, *P* representing the demographic factor, *A* representing the economic development factor, *T* representing the technical factor, and *α* and *e* representing the constant coefficients and random disturbance items, respectively.

(3) $$I_{it}=\alpha_{i}P_{it}^{b}A_{it}^{c}T_{it}^{d}e_{it}$$

Considering the adoption of the linear equation form, replacing the dependent variable with the outcome variable in this study, and including the core independent PPPST variables and other important control variables, the extended model form of this paper can be expressed as Function (4), which constitutes the specific empirical analysis model, where *WPPP* is the core independent variable. According to the variables initially included in the IPAT model and referring to related research [46], in this study, the following main variables were selected: population size (*LNPOP*), economic development level (*LNPGDP*), technological innovation level (*TECH*), industrial structure (*INDSTR*), financial development (*FINANCE)*, foreign capital proportion (*FDIR*), and fiscal condition (*FISCAL*). Moreover, *ρ* is the constant term, *c_i_* and λ_t_ are the individual and the year fixed effects, respectively, and *ε* is the random disturbance term.

(4) $$\begin{array}{l} LNSW_{it}=\rho +\delta \times WPPP_{it}+\beta_{1}\times LNPOP_{it}+\beta_{2}\times LNPGDP_{it}\\ +\beta_{3}\times TECH_{it}+\beta_{4}\times INDSTR_{it}+\beta_{5}\times FINANCE_{it}\\ +\beta_{6}\times FDIR_{it}+\beta_{7}\times FISCAL_{it}+c_{i}+\lambda_{t}+\varepsilon_{it} \end{array}$$

The relevant variables are described below.

Dependent variable: sewage disposal (*LNSW*). This study used the urban sewage disposal provided in the Urban Construction Statistical Yearbook as its measurement (unit: 10,000 cubic meters). Considering the consistency with the logarithm of the STIRPAT model and the asymmetric nature of the distribution of the value itself, this study used its logarithmic form.

Core independent variable: whether a city has initiated PPPSTs (*WPPP*). This study mainly used a dummy variable form for its measurement, which is a convenient way of setting the DID model. The cities were distinguished into a treatment group and a control group based on whether a city initiated PPPSTs. In addition, this study also considered the difference in the scale of the investment amount of PPPSTs and clustered the project capital investment scale to the city level to obtain a continuous variable tagged as *LNWPPPI*, which is the logarithm of the investment. The *WPPP* variable is replaced by *LNWPPPI* later in this paper to construct a continuous DID model.

Control variables: (a) Population size (*LNPOP*)—measured by the logarithm of the city’s total population at the end of the year; this variable was chosen because, in general, the larger the population, the greater the demand for sewage disposal, which is important concerning the population’s domestic water demands. (b) Economic development (*LNPGDP*)—measured by the logarithm of per capita GDP. Controlling the difference in urban economic development levels can effectively feedback the change in sewage disposal demand brought about by the agglomeration of economic activities, which is also one of the fundamental variables of the IPAT model [46]. (c) Technological factors (*TECH*)—the technological progress variable, included because it is considered one of the developmental factors with wide-ranging effects [7]. In this paper, two variables were selected as inputs: the proportion of science and technology expenditure in GDP (*TCHR*) and the proportion of education expenditure in GDP (*EDUR*). (d) Industrial structure (*INDSTR*)—the industrial structure can reflect the transformation of the city’s overall economic activities and reflect a specific industrial agglomeration situation, thereby indirectly reflecting the utilization and dependence of economic activities on natural resources such as water resources [47,48]. Drawing on relevant analysis, this study also used two indicators to measure the structures; these are the proportions of the secondary and tertiary industries in GDP (*ISR*), and the ratio of the tertiary industry to the secondary industry (*ISODD*). One reflects the degree of non-agriculturalization, and the other reflects the degree of industrial sophistication. (e) Foreign direct investment (*FDI*)—measured as the proportion of foreign direct investment in GDP. This variable is introduced because of the importance of its productivity spillover effect [49]. (f) Finance development (*FINANCE*)—measured as the ratio of the balance of deposits and loans of financial institutions to GDP. One of the reasons for applying the PPPs is the financing problem, which is also the reason for introducing financial-related variables in this study. (g) Fiscal factors (*FISCAL*)—in China, public service infrastructure is often covered by local finance, so the financial situation of cities cannot be ignored. This study introduced two variables, the proportion of fiscal revenue in GDP (*GFR*) and fiscal expenditure in GDP (*GFR*), to reflect the city’s financial situation.

### 3.3. Suitability Test of DID Model

The rationality of the DID method is conditional. While it requires exogenous policy interventions, it should also satisfy certain assumptions because the DID framework can be used as a better instrumental variable to identify causal relationships only when the policy is exogenous. The model can only attribute the effect to the core explanatory variable if it satisfies parallel trends and when no other policy effects are omitted. To ensure the credibility and stability of the estimation results, this study sequentially tested the important identification hypotheses.

#### 3.3.1. Parallel Trend Test

An important premise for the validity of the DID method is the common trend assumption; that is, if cities in the treated group do not initiate PPPSTs, then the changing trend of sewage disposal should be parallel with that in the control group. There have long been critical discussions about the test for parallel trends [42,50]. Accordingly, this study adopted an event study model to capture the dynamic impact of PPPSTs on urban sewage disposal by introducing event time *τ*; see Function (5).

(5) $$\begin{array}{l} \ln sewage_{it}=\rho +\sum_{k=-m}^{-2}\theta_{k}\times treat\times 1\{\tau =k\}+\sum_{k=0}^{q}\theta_{k}\times treat\times 1\{\tau =k\}\\ +\beta_{1}\times LNPOP_{it}+\beta_{2}\times LNPGDP_{it}\\ +\beta_{3}\times TECH_{it}+\beta_{4}\times INDSTR_{it}+\beta_{5}\times FINANCE_{it}\\ +\beta_{6}\times FDIR_{it}+\beta_{7}\times FISCAL_{it}+c_{i}+\lambda_{t}+\varepsilon_{it} \end{array}$$

where 1{*τ* = *k*} is a series of dummy variables, representing the *k*-th year when the urban PPPST was initiated; for example, *τ* = 0 indicates the current year when the city first initiates PPPSTs, *τ* = −2 means two years before the initiation. The event study’s parameter is *θ_k_*, which represents the difference in sewage disposal between the treated group and the control group in the *k*-th year of the invention. If the trend of *θ_k_* during the *k* < 0 is relatively gentle and not significantly different from zero, it proves that the parallel trend hypothesis is met. On the contrary, if the coefficients have significant values when *k* < 0, there are significant differences between the treated group and the control group before the policy is implemented, which does not meet the parallel trend hypothesis.

#### 3.3.2. Placebo Test

Another concern regarding the identification assumptions of the DID method is the effect of unobservable characteristics that vary over time on the estimates. The characteristics of cities are very different and challenging to identify fully. Although the previous identification controls the impact of all urban characteristics that do not change with time on sewage disposal by adding city fixed effects, some characteristics may change over time, thereby affecting the identification hypothesis. That is, the effects of other unobservable variables need to be evaluated. This study adopted an indirect placebo test, which is widely used in the relevant literature [51,52].

According to the empirical analysis model in this paper, the estimation of the coefficient *θ* can be obtained by Function (6).

(6) $$\hat{\theta }=\theta +\gamma \times \frac{\mathrm{cov}(treat,\varepsilon |W)}{\mathrm{var}(treat|W)}$$

where *W* includes all other control variables and fixed effects, and *γ* is the influence of non-observed factors on the coefficient of the core independent variable. If *γ* = 0, the unobserved factor will not affect the estimation result. It proves that *θ* hat is unbiased, but this cannot be directly verified. Therefore, this study adopted an indirect method of the placebo test, the logic of which is to find a false variable that theoretically does not affect the outcome variable to replace the *WPPP* in the original model. Since the false variable is randomly generated, *θ* hat would be zero; else, if this wrongly estimated variable affects the result, that is, *θ* hat is not zero, it proves that the estimated equations in this paper may be biased, indicating that other unobserved characteristics can affect the estimated results.

### 3.4. Data Sources

The data in this paper were taken from the PPP project management database of the Government Social Capital Cooperation Center of the Ministry of Finance of the People’s Republic of China, the Chinese Urban and Rural Construction Database of EPS DATA, and the Chinese City Database EPS DATA. Here, EPS DATA refers to the Economy Prediction System, which can be accessed at https://www.epsnet.com.cn/index.html#/Index (accessed on 1 March 2022). This data platform provides researchers with simple, professional, and high-quality data services, with an average annual data download of 1 billion data points, providing solid data support for academic research in related fields, fully reflecting the academic value and authority of EPS DATA.

Specifically, first, the dependent variable sewage disposal was taken from the “sewage disposal (10,000 cubic meters)” indicator in the Chinese Urban and Rural Construction Database of EPS DATA, which is collected from the Ministry of Housing and Urban-Rural Development of the People’s Republic of China.

Second, the PPP project-related data were taken from the “Project Library Information Disclosure” column provided by the China Public-Private Partnerships Center website http://www.cpppc.org/home.jhtml (accessed on 3 September 2021). For this study, a web information crawling program was written using STATA 16.0 (StataCorp LLC, College Station, TX, USA), which can be requested by contacting the author, and was used to obtain all PPP project information in the library from 2009 to the end of 2021. The information includes the address where the projects are located, their industry (primary or secondary industry), the total investment amount of each project, the time of initiation, the stage, the cooperation period, and the operation modes. According to the secondary industry to which the PPP project belongs, it is easy to identify whether the project is a sewage treatment project. Thus, the city-level *WPPP* variable can be further constructed according to the address of each city. At the same time, based on the total investment amount of the project, in this study, the capital scale of PPPSTs was summed by city to obtain the *LNWPPPI* variable.

Thirdly, the basic data indicators related to the control variables were taken from the Chinese City Database of EPS DATA, which has compiled the indicator information in the Chinese City Statistical Yearbook over time in a more detailed manner, thus providing a wealth of urban characteristics variables. Table 2 presents the descriptive statistics of the relevant variables.

**Figure 5:**
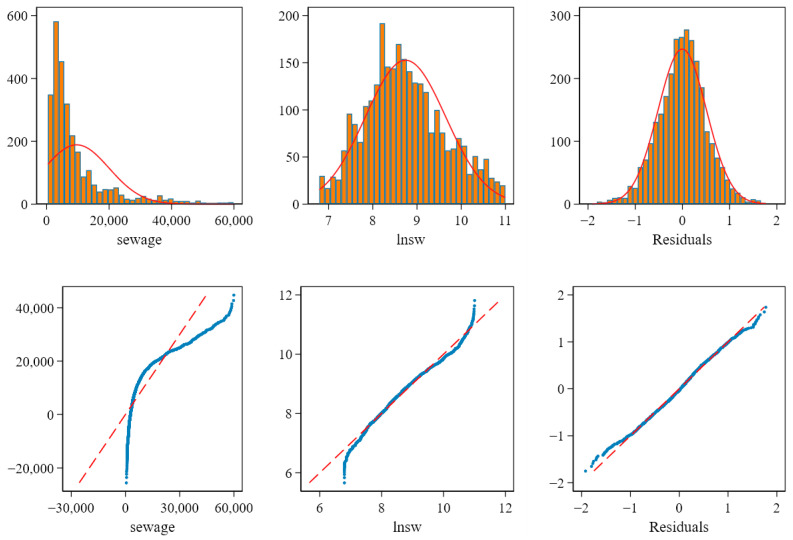
The normality test of the sewage disposal, *LNSW,* and the residual after regression. Note: The author drew figures via STATA 16.0 (StataCorp LLC, College Station, TX, USA).

Regarding the normality of variables, the skewness and kurtosis indexes can be used to make a preliminary judgment. The related results in Table 2 show that some variables have a distribution bias. In this condition, to improve the reliability of the empirical analysis, the normality of dependent variables is further discussed. The reason for this is that, first, directly transforming all variables, such as via logarithmic transformation or square root transformation, will significantly affect the interpretation of economic meaning; second, regression analysis mainly requires the normality of the dependent variable or, more accurately, the residual according to Gauss–Markov’s theorem. Therefore, it is worth paying attention to whether the residual after the regression is subject to a normal distribution to allow a reasonable statistical inference. Therefore, this study tested the normality of the sewage discharge variable and the residual variable after the regression using the histogram and qnorm plot (i.e., the plot of quantiles of a variable against quantiles of the normal distribution) to expand the test for the intuitive observation results. The test results are shown in Figure 5.

Where the reference line corresponds to the standard normal distribution, the information shown in the figure shows that the level variable of urban sewage disposal has an evident right-bias phenomenon. However, the *LNSW* variable is close to the normal distribution, and the residual variable after regression is closer to the standard normal distribution. This is verified in both histogram and qnorm plots. This study also applied the skewness/kurtosis tests for normality. The results show that the residual’s *p*-value of the skewness statistic is 0.3143. There is no pronounced asymmetry, but the kurtosis (*p* < 0.000) result shows that the residual is different from the standard normal. Given the symmetry of its distribution, the *t*-test can be a good substitute for the Z-test, based on the statistical test that is launched. In general, the model in this paper is in line with the assumption basis of reasonable statistical inference.

**Figure 6:**
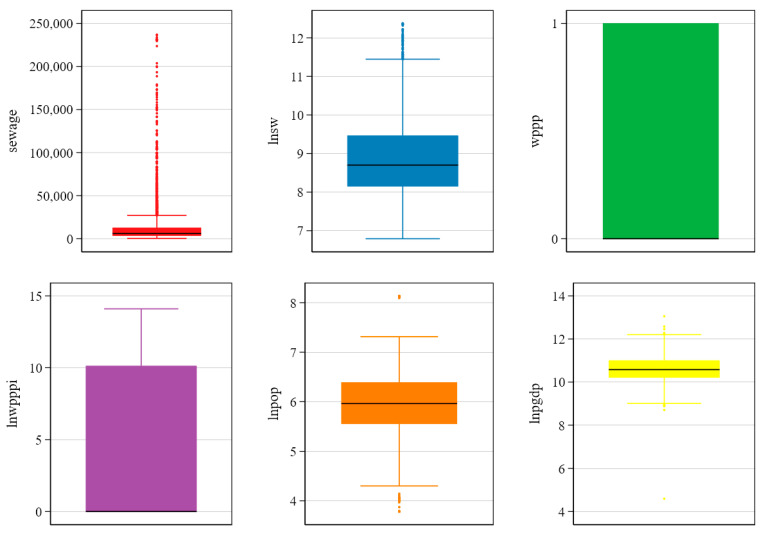
Outlier detection of variables. Note: The author drew figures via STATA 16.0 (StataCorp LLC, College Station, TX, USA).

Regarding outliers, the outside values of several key variables are shown in box plots; see Figure 6. The results show that the raw sewage data are scattered because the data are from different years and cities. There are significant differences in the development stage and scale of Chinese cities. The development of Chinese cities varies significantly over time, so the statistical outliers of the level value variables are more significant. According to the box plot of *LNSW*, when the logarithm of sewage is taken, the outlier problem is significantly controlled. Furthermore, the core independent variables *WPPP* and *LNWPPPI* have no significant outliers. In contrast, *LNPOP* and *LNPGDP* have only a small number of outliers, suggesting that the variable outliers examined in this study are not significant and can be better analyzed in the future.

## 4. Results

### 4.1. Basic Results

Robust standard errors were applied to the econometric estimation results in this paper. To clarify the reasons for adopting robust standard errors, this study conducted some tests of the homoscedasticity properties of the data and variables. Specifically, two main methods were used: (a) the homoscedasticity of the data—the analysis was carried out by the two-sample variance-comparison test using the groups method; (b) heteroscedasticity test—the Breusch–Pagan/Cook–Weisberg test for the heteroskedasticity test method (BPCW) was applied. The results indicate that, for the variable *LNSW*, under the condition of the treatment group and the control group, the F test for equality of variances performed at 1.0875 (*p* = 0.101), and the robust F test performed at 2.603 (*p* = 0.107), both of which are not significant, indicating that there is no significant variance difference in the data between the two groups of samples. The BPCW test exhibited a chi-square value equal to 3.82 (*p* = 0.051). That is, there is a specific heteroscedasticity problem near the critical level of 5%. For rigor, this study applied robust standard errors in the regression process.

This study also conducted a collinearity assessment on related variables to identify redundancy between variables. Several collinearity diagnostic measures, including VIF, tolerance, and R-squared, were used to check for multicollinearity among variables. Results are shown in Table 3. In general, values of VIF > 10, or tolerance < 0.2, or R^2^ > 0.9, suggest consideration of multicollinearity among the independent variables. According to the data analysis results, the mean value of the variable VIF is 2.40, which is significantly smaller than 10, the maximum value of VIF for variables is 3.79, which is also less than 10, and the calculated Sqrt VIF mean value of 1.52 is also significantly less than 10; the minimum value of each variable’s tolerance is 0.2637, which is also larger than the usual critical value, and the mean value of tolerances is 0.503, which is significantly higher than the judgment value of 0.2; similarly, the mean R^2^ is 0.4970, and the R^2^ of each variable is less than 0.9. Based on the above results, it is suggested that there is no significant collinearity, so, in this study, it was believed that the data and variables used do not have serious multicollinearity problems.

Next, this study estimated the DID model with different settings of methods, such as OLS (ordinary least squares), RE (random effect panel data model), and FE (fixed effect panel data model). The values of the coefficient *δ* show statistically significant negative results under the three methods, which are −0.080 (*p* = 0.002), −0.039 (*p* = 0.004), and −0.036 (*p* = 0.004), respectively, all of which are significant at the 1% level. At the same time, the Hausman test value of whether the model should be in the fixed effects or random effects form is 386.029 (*p* < 0.001), indicating that it is more appropriate to support the FE model. This enhances the rationality of using the two-way fixed-effects condition to estimate the DID model in this study.

The results of the control variables through Model (3) are shown in Table 4. After controlling for the city fixed effect, the coefficient of *LNPOP* is 0.085 (*p* = 0.213), which is not statistically significant. In contrast, the results in (1) and (2) are significantly larger, and are 0.714 (*p* < 0.001) and 0.537 (*p* < 0.001), respectively. In terms of econometric estimation, this reflects the differences in the results of mixed regression, and between-group and within-group estimators under different assumptions, and confirms the need to control the urban fixed effect. This result also shows that the population change is not the main reason for the change in sewage disposal for cities. The coefficient of the urban economic development level is 0.117 (*p* < 0.001), which is significantly positive. Every 1% of per capita GDP results in a 0.117% increase in sewage disposal. Therefore, it can be seen that economic development is an important reason for the increase in sewage disposal. Investment in science and technology and education showed positive relationships with sewage disposal. In addition, the non-agricultural process of the industrial structure also significantly increased the amount of sewage disposal, since the coefficient of ISR is 0.009 (*p* < 0.001) is statistically significant. Foreign direct investment, financial development, and local government fiscal revenue and expenditure do not show statistical significance in the sample of this study.

### 4.2. Results of Suitability Test of DID Model

#### 4.2.1. Results of Parallel Trend Test

**Figure 7:**
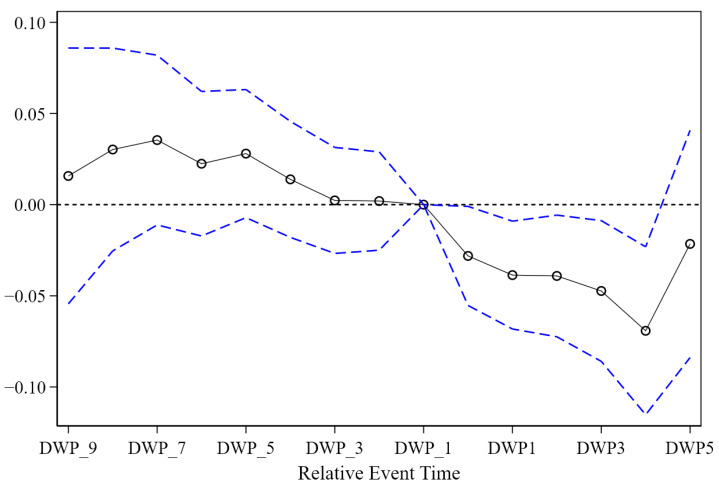
Parallel trend test under the event studied. Note: The author drew the figure via STATA 16.0 (StataCorp LLC, College Station, TX, USA).

The parallel trend test results are reported in Figure 7, specifically, when *τ* = −1 is used as the reference period. This study used *DWP_k* to represent the dummy variable corresponding to when *k* is negative, and *DWPk* to represent the corresponding dummy variable when *k* is positive. In the interval of *k* < 0, the values of *θ_k_* are not significant, indicating that there is no significant difference between the cities that initiated PPPSTs and the cities in the control group before the initiation. However, starting from *k* = 0, *θ_k_* begins to decrease significantly, indicating that the construction of PPPSTs significantly reduced urban sewage disposal.

#### 4.2.2. Results of Placebo Test

**Figure 8:**
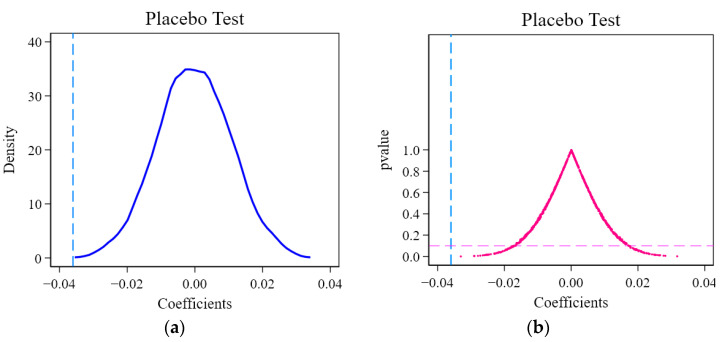
Placebo test: (**a**) coefficient distribution, and (**b**) *p*-value distribution. Note: The author drew figures via STATA 16.0 (StataCorp LLC, College Station, TX, USA).

Specifically, this study randomly generated a list of cities that have initiated PPPSTs, thereby generating an incorrect estimate of *θ_placebo_*. After repeating this process 1000 times, 1000 *θ_placebo_* values were generated accordingly. Figure 8 depicts the distribution of random coefficients for the placebo test. It is not difficult to see that the placebo coefficients are distributed near zero, and the distribution is approximately normal. Thus, it can be deduced that γ = 0, which is in line with the prediction of the placebo test, thus proving that the unobserved regional features hardly have a severe impact on the estimation results in this model. Thus, the results of the basic models in this paper are robust.

### 4.3. Extended Study under Continuous Variables

Next, two continuous variables were used to apply continuous DID models, namely, the duration and the investment amount of PPPSTs. Based on the introduction of continuous variables, this study was also concerned about the nonlinear structure under different conditions; hence, the estimation models, including the quadratic term of the continuous variables, were also estimated.

The results are reported in Table 5. Panel A shows the estimation of the duration of PPPSTs as a continuous variable. The duration coefficient is −0.007 (*p* = 0.097), indicating that the impact on sewage disposal gradually increases over time under the assumption of linear impact. In the model containing the quadratic term of duration, the coefficient of the horizontal term is −0.026 (*p* = 0.001), and the coefficient of the quadratic term is 0.003 (*p* = 0.003), which is statistically considered to have a significant U-shaped relationship. Considering the duration of cities in the sample is mostly less than seven years, the comprehensive effect is still negative. That is, PPPSTs can reduce sewage disposal.

Panel B shows the results when the investment amount of PPPSTs is used to measure the policy intervention variable. Its coefficient is −0.002 (*p* = 0.034) and is statistically significant at the 5% level. Furthermore, when the quadratic term is included, the coefficient of the horizontal term is −0.022 (*p* < 0.001), and that of the quadratic term is 0.002 (*p* < 0.001). This result is similar to that of duration, and a reduction effect at the overall level can be calculated. Therefore, this paper confirms the beneficial effect of PPPSTs in reducing urban sewage disposal by adopting continuous DID models.

## 5. More Analysis

### 5.1. Does the Operating Mode of PPPSTs Matter?

PPP projects usually have different operating modes and face different management risks, which lead to differences in the local government’s ability to control the project or the level of project operation efficiency. As mentioned earlier, it is of interest to study whether there are differences in this paper’s thematic effects for different types of operations. Therefore, this subsection discusses this.

To facilitate subsequent model processing, this study used the BOT type as the benchmark to construct a mode of operation (*MOP*) variable to measure the proportion of the BOT type of the operating modes in all PPPSTs for each city. The moderating effect of the variable was then identified by interactively multiplying *MOP* with the core independent variables. In Section 5, all analyses are carried out in this manner.

The results in Table 6 show that *MOP* of PPPSTs results in heterogeneity in the effects. The coefficient of *WPPP* × *z_MOP* is −0.050 (*p* = 0.008), and that of *z_LNWPPPI* × *z_MOP* is −0.009 (*p* = 0.059), both of which are negative and significant. This shows that different operating modes affect the impact of PPPSTs on urban sewage disposal reduction to a certain extent.

### 5.2. Does Fiscal Decentralization Matter?

Will the city’s fiscal situation affect wastewater disposal? Fiscal-related considerations are crucial reasons why cities choose PPPSTs. The study was also interested in the impact of government behavior choices on sewage disposal under different fiscal conditions. To this end, according to the relevant information on urban fiscal expenditure, this study constructed the fiscal decentralization index of the urban government (*CZFQ*). The calculation formula is shown in Function (7), in which *PCFE_C*, *PCFE_P*, and *PCFE_N* represent the per capita fiscal expenditure of the sample city, province, and country, respectively.

(7) $$CZFQ_{it}=\frac{PCFE\_C_{it}}{PCFE\_C_{it}+PCFE\_P_{it}+PCFE\_N_{it}}$$

This study used two approaches to examine whether fiscal decentralization has a moderating effect on urban wastewater disposal. One is to use the fiscal decentralization variable *CZFQ* to multiply the core independent variables (See Panel A in Table 7); the other is to divide the cities into three types of fiscal decentralization—low, medium, and high—and use the dummy variables to multiply the core independent variables (see Panel B in Table 7).

The results show that, in Panel A, the coefficient of *WPPP* × *z_CZFQ* is −0.022 (*p* = 0.658), and the coefficient of *z_LNWPPPI* × *z_CZFQ* is −0.003 (*p* = 0.776), both of which are negative but statistically insignificant. Thus, there is not enough evidence to show that the state of fiscal decentralization significantly affects the impacts of PPPSTs on sewage disposal. To further verify the reliability of this result, Panel B reports the effect differences under the conditions of high and low classification using fiscal decentralization. In the results, the coefficient of *WPPP* × *CS2* is 0.009 (*p* = 0.610), the coefficient of *WPPP* × *CS3* is 0.031 (*p* = 0.112), the coefficient of *LNWPPPI* × *CS2* is 0.001 (*p* = 0.781), and the coefficient of *LNWPPPI* × *CS3* is 0.004 (*p* = 0.240). Therefore, it can be seen that the coefficients of the interaction terms are not significant. Thus, it is believed that the degree of fiscal decentralization has no significant moderating effect on the relationship between the initiation of PPPSTs and urban sewage disposal.

### 5.3. Does National Demonstration Project Matter?

In China, there are different categories of PPPSTs, such as national demonstration projects, provincial demonstration projects, and municipal demonstration projects. One of the more concerning questions in this paper is, will city-initiated projects perform better when they are labeled as demonstration projects? Intuitively, based on the logical deduction of project governance, local governments have incentives to implement these projects more strictly when they initiate national demonstration projects. To test this hypothesis, the dummy variable of whether there are national demonstration projects was introduced into the model using interactions. The results are shown in Table 8.

The results show that the coefficient of *WPPP* × *NATION* is −0.034 (*p* = 0.041), and the coefficient of *z_LNWPPPI* × *NATION* is −0.008 (*p* = 0.043), both of which are negative and statistically significant at the 5% level, which means that, when there are national demonstration projects in a city, the impact of PPPSTs on reducing sewage disposal is more effective.

## 6. Discussion

The problem of water pollution caused by urban sewage disposal is also of wide concerned in China, and the PPP approach has increasingly been adopted in the sewage treatment field. Moreover, the relevant methods and demonstrations for improving the efficiency of water supply and wastewater treatment in different countries have been examined, providing a sound theoretical background for discussing the results of this paper and explaining the reasons for taking PPPSTs as the research object.

This study found that the initiation of PPP sewage treatment projects has a statistically significant reduction effect on urban sewage disposal. This effect is still valid when the core independent variable is characterized by duration or investment amount, rather than the binary dummy variable. According to the empirical results, compared with the cities without PPPSTs, sewage disposal decreased by 3.6%, which is statistically significant at the 1% level. This result is obtained under the two-way fixed effect model, compared with the estimations of the OLS model and the RE model, which show reductions of 8.0% and 3.9%, respectively. The coefficient value is relatively small, but this study considered it to be more credible, and it answers the question of whether PPPSTs can help reduce urban wastewater disposal.

Regarding the applicability tests of the analytical model in this paper, two main questions were explored. One is whether the ex ante development trends of the cities that initiated PPPSTs and those that did not initiate them are consistent, which was examined through an event study. According to the estimation results, it is clear that the estimated coefficients between groups in this paper are not statistically significantly different from zero. That is, the basic assumption of parallel trends is considered to be satisfied. The other was examined via the construction of a placebo test to determine whether other unobservable characteristics affect the change in urban sewage disposal. The focus excludes whether it is caused by other policies rather than the construction of PPPSTs. The test results support the passing of the placebo test, so the analysis results in this paper are highly credible. Using continuous variables to replace the core independent variable, this paper discusses the effects of PPPSTs’ duration and investment scale. The results can still reflect the reduction effect of PPPSTs on sewage disposal. In the models with the quadratic term, the coefficient results show that reduction effects first increase and then weaken, with changing marginal effects.

In addition, this study uniquely analyzed some mechanisms that may be of interest, which is one of the essential features of this study that distinguishes it from other research. First, it explored whether there are differences in effects under different operating modes. The empirical results show that, when the interaction term of the core explanatory variable and the operating mode is introduced, its coefficient is significantly negative, which means that the operating mode of the project presents a more significant effect on reducing sewage disposal. In Chinese cities, the BOT type is the primary mode of operation adopted, indicating that when the local government adopts PPPSTs, there is a certain amount of effort in the contract arrangement. This positive management attitude has improved the management of sewage disposal. Operating modes may lead to different incentive mechanisms for private suppliers. Therefore, this study aper suggests that, in the management contract of PPPSTs, attention should be paid to the balance of interests of multiple parties [12], to further mobilize the investment and operation enthusiasm of private suppliers, and improve the performance of projects.

Second, it discusses the effect of fiscal factors on urban sewage disposal. Through a similar moderation effect analysis method, this study did not find statistically significant evidence to support the idea that the local government’s fiscal status significantly affects the impact of PPPSTs on sewage disposal. The possible reason for this is that the decentralization of urban fiscal spending mainly reflects the distribution of executive power and financial power between the local government and the higher-level government. Therefore, it focuses more on development, and thus mainly affects related development goals rather than investment in basic municipal facilities; as a result, it has little effect on urban sewage disposal. To discuss the estimation result more robustly, this study also constructed dummy variable multiplication terms by distinguishing the cities into three decentralization status groups of low, medium, and high. The results still indicate that the fiscal decentralization status does not significantly affect the intervention impact of PPPSTs on sewage disposal. This enhances the demonstration effect of launching PPPSTs, which may be one of the important reasons for reducing sewage disposal.

Third, it analyzed the impact of national-level demonstration projects in cities. According to the results presented in Section 5.3, the coefficient values of the multiplication term of the core independent variable and the national demonstration project variable are significantly negative, which means that cities show a stronger constraint on sewage disposal when they have national demonstration projects. This paper believes that there are two reasons for this. First, the national demonstration projects are more stringent in supervision and may substantially impact sewage disposal. Second, to achieve a good reputation, local governments often pay attention to the socio-economic effects of such projects, thus leading to the moderating effect of demonstration projects on reducing sewage disposal. In addition, the government’s performance monitoring and assessment of PPPST projects encourage private suppliers to avoid penalties for failing to meet the project objectives, giving private suppliers incentives to improve efficiency.

The management and control of urban sewage disposal and sewage treatment are basic requirements of ecological civilization. PPPs, which are widely used in this field, provide local governments with a more relaxed approach to financing. Moreover, the integration of private enterprises is conducive to improving efficiency and forming a demonstration effect. Previous studies mainly focus on risk assessment [4], asset securitization (ABS) in financing models [53], and financial feasibility [54] of PPPs. This study, in contrast, used urban sewage disposal as the dependent variable to explore the effect of PPPSTs on sewage disposal reduction. This effectively supplements the empirical evidence at the city level in this field and supports the successful application of PPPs in the field of sewage treatment in China.

Moreover, PPP projects are not unique to China. For example, the PPP approach is widely used in countries along the Belt and Road, such as in transportation and public service infrastructure. Therefore, the discussion and implications presented in this paper can also provide a reference for other countries to conduct related assessments to a large extent.

## 7. Conclusions

The study provides primary empirical evidence to clarify the relationship between PPP and wastewater disposal governance. The Chinese PPP project data were matched to the city construction and development data, and a large and well-represented sample dataset was obtained. Using the panel data of 267 prefecture-level cities from 2009 to 2020, a DID model was constructed in this study under the framework of counterfactual analysis. This model identified the impact of the shock of implementing PPPSTs on urban sewage disposal.

The results show that PPPSTs contribute to the reduction in sewage disposal. In the benchmark regression, the *WPPP* coefficient was −0.036 (*p* = 0.004), which is significant at the 1% level, when the core independent variable was replaced by *DURATION* or *LNWPPPI*, and the quadratic term for the continuous variable was introduced. When identifying the moderating effects of operating modes, fiscal decentralization, and the national demonstration projects, as shown in Section 5, the results all support the reduction effect of the initiation of PPPSTs on urban wastewater disposal. The relevant conclusions will help to further clarify the rationality and practical benefits of PPPs in sewage treatment, and have meaningful significance for urban management. In addition, the discussion and implications presented in this paper can also provide a reference for other countries since PPPs are widely applied around the world.

Some possible limitations are that, first, although the impact of PPPs at the city level has been identified as accurately as possible in this paper, following a more rigorous logic, the impact of PPPs occurs primarily at the micro-enterprise level. The identification of the effect at the city level has certain aggregate and coverage effects. These effects reflect the comprehensive impact of implementing PPPSTs in a local geographic location of the city on the overall sewage disposal of the entire city. Second, Chinese cities have different economic, geological, and ecological characteristics or are in different stages of development. Due to this heterogeneity, it is difficult to make a single policy recommendation. Spatial and cultural differences must be considered, and, at the same time, a more detailed examination of their internal mechanism between the variables must be undertaken. Therefore, future research works on PPPSTs can be deepened by (a) investigating individual enterprises at the level of micro-sample data to explore the micro-mechanisms of relative effect changes; and (b) introducing spatial and cultural dependencies in model approaches, such as the spatial lag model and spatial heterogeneity model of spatial measurements, to closely explore the spatial and temporal differences of sewage treatment efficiency. This paper believes that expanding the study of micro-individual and spatial perspectives will help address the problems associated with PPP sewage treatment.

## Figures and Tables

**Table 1:** Description of the meaning of PPPST operation modes.

Abbreviation	Implication
BOO	Build–Own–Operate
BOT	Build–Operate–Transfer
TOT	Transfer–Operate–Transfer
ROT	Renovate–Operate–Transfer
OM	Operations and Maintenance
MC	Management Contract
LOT	Lease–Operate–Transfer
BLOT	Build–Lease–Operate–Transfer
BLMT	Build–Lease–Maintain–Transfer

**Table 2:** Variable settings and descriptive statistics.

Variables	A Brief Description of the Setting	Obs	Mean	SD	Min	Median	Max	Skewness	Kurtosis
*LNSW*	The logarithm of sewage disposal	3204	8.86	1.063	6.79	8.70	12.37	0.45	2.13
*WPPP*	Have PPPSTs (yes = 1; no = 0)	3204	0.34	0.474	0.00	0.00	1.00	−0.08	1.01
*LNWPPPI*	The logarithm of PPPSTs’ investment	3204	3.75	5.280	0.00	0.00	14.10	0.01	1.10
*LNPOP*	logarithm of the total population	3204	5.94	0.643	3.78	5.96	8.14	−0.29	3.31
*LNPGDP*	logarithm of per capita GDP	3204	10.61	0.613	4.60	10.58	13.06	−0.23	4.33
*TCHR*	Technology spending as a share of GDP	3204	0.26	0.244	0.01	0.18	4.15	1.27	4.68
*EDUR*	Education spending as a share of GDP	3204	3.21	1.416	0.34	2.90	13.03	0.47	2.46
*ISR*	Secondary and tertiary industries as a share of GDP	3204	87.73	7.857	50.10	88.80	100.00	−1.42	4.61
*ISODD*	The ratio of tertiary industry to secondary industry	3204	0.94	0.509	0.18	0.83	5.15	2.01	7.12
*FDIR*	Foreign direct investment as a share of GDP	3204	0.33	0.446	0.00	0.21	11.48	1.04	4.63
*FINANCE*	Deposit and loan balance as a percentage of GDP	3204	2.32	1.169	0.59	1.99	21.30	0.71	2.95
*GFR*	Deposit and loan balance as a percentage of GDP	3204	0.47	0.222	0.06	0.44	1.54	0.40	5.87
*GFR*	Fiscal spending as a percentage of GDP	3204	0.18	0.085	0.04	0.16	1.49	0.53	2.02

**Table 3:** Collinearity assessment.

Variable	VIF	SQRT VIF	Tolerance	R-Squared
*WPPP*	1.51	1.23	0.6634	0.3366
*LNWPPPI*	1.27	1.13	0.7890	0.2110
*LNPOP*	3.51	1.87	0.2851	0.7149
*LNPGDP*	1.41	1.19	0.7069	0.2931
*TCHR*	3.35	1.83	0.2987	0.7013
*EDUR*	2.95	1.72	0.3389	0.6611
*ISR*	1.9	1.38	0.5275	0.4725
*ISODD*	1.13	1.06	0.8845	0.1155
*FDIR*	2.01	1.42	0.4985	0.5015
*FINANCE*	3.79	1.95	0.2637	0.7363
*GFR*	3.61	1.9	0.2772	0.7228
Mean	2.40	1.52	0.5030	0.4970

**Table 4:** Basic regression results.

	(1)				(2)				(3)			
	*LNSW*				*LNSW*				*LNSW*			
	Coef.	t	p	95% CI	Coef.	t	p	95% CI	Coef.	t	p	95% CI
*WPPP*	−0.080 ***	−3.132	0.002	−0.130, −0.030	−0.039 ***	−2.890	0.004	−0.065, −0.012	−0.036 ***	−2.888	0.004	−0.061, −0.012
*LNPOP*	0.714 ***	41.271	*p* < 0.001	0.680, 0.748	0.537 ***	12.826	*p* < 0.001	0.455, 0.619	0.085	1.247	0.213	−0.049, 0.219
*LNPGDP*	0.350 ***	11.576	*p* < 0.001	0.290, 0.409	0.188 ***	7.100	*p* < 0.001	0.136, 0.240	0.117 ***	4.616	*p* < 0.001	0.067, 0.167
*TCHR*	0.272 ***	5.656	*p* < 0.001	0.178, 0.367	0.051 *	1.915	0.055	−0.001, 0.102	0.053 **	2.084	0.037	0.003, 0.102
*EDUR*	−0.207 ***	−16.209	*p* < 0.001	−0.232, −0.182	−0.015	−1.630	0.103	−0.033, 0.003	0.024 ***	2.657	0.008	0.006, 0.041
*ISR*	0.019 ***	8.768	*p* < 0.001	0.015, 0.023	0.019 ***	8.142	*p* < 0.001	0.015, 0.024	0.009 ***	3.888	*p* < 0.001	0.005, 0.014
*ISODD*	0.269 ***	10.070	*p* < 0.001	0.216, 0.321	0.115 ***	5.254	*p* < 0.001	0.072, 0.158	0.005	0.251	0.802	−0.037, 0.048
*FDIR*	0.043 *	1.830	0.067	−0.003, 0.089	−0.014	−1.268	0.205	−0.035, 0.008	−0.013	−1.285	0.199	−0.033, 0.007
*FINANCE*	0.177 ***	14.764	*p* < 0.001	0.153, 0.200	0.027 ***	3.655	*p* < 0.001	0.012, 0.041	−0.002	−0.264	0.792	−0.015, 0.012
*GFR*	0.779 ***	8.994	*p* < 0.001	0.609, 0.949	0.280 ***	4.539	*p* < 0.001	0.159, 0.402	0.040	0.677	0.499	−0.077, 0.157
*GFR*	0.787 ***	3.574	*p* < 0.001	0.355, 1.219	0.135	1.273	0.203	−0.073, 0.342	−0.018	−0.180	0.857	−0.213, 0.177
Obs.	3204				3204				3204			
R-sq	0.748				0.669				0.444			
Model	OLS				RE				FE			
City_FE	YES				YES				YES			
Year_FE	NO				NO				YES			
Cities					267				267			
Hausman						386.029 ***						

Note. *** *p* < 0.01, ** *p* < 0.05, * *p* < 0.10.

**Table 5:** Estimation results under continuous DID models.

	(1)				(2)			
	*LNSW*				*LNSW*			
	Coef.	t	p	95% CI	Coef.	t	p	95% CI
Panel A								
*DURATION*	−0.007 *	−1.658	0.097	−0.016, 0.001	−0.026 ***	−3.384	0.001	−0.040, −0.011
*DURATION^2*					0.003 ***	2.967	0.003	0.001, 0.006
Obs.	3204				3204			
R-sq	0.443				0.445			
Model	FE				FE			
Control	YES				YES			
City_FE	YES				YES			
Year_FE	YES				YES			
Cities	267				267			
Panel B								
*LNWPPPI*	−0.002 **	−2.117	0.034	−0.005, −0.000	−0.022 ***	−4.047	*p* < 0.001	−0.033, −0.012
*LNWPPPI^2*					0.002 ***	3.681	*p* < 0.001	0.001, 0.003
Obs.	3204				3204			
R-sq	0.443				0.446			
Model	FE				FE			
Control	YES				YES			
City_FE	YES				YES			
Year_FE	YES				YES			
Cities	267				267			

Note. *** *p* < 0.01, ** *p* < 0.05, * *p* < 0.10.

**Table 6:** The impact of the operating mode of PPPSTs.

	(1)				(2)			
	*LNSW*				*LNSW*			
	Coef.	t	p	95% CI	Coef.	t	p	95% CI
*WPPP*	−0.010	−0.635	0.525	−0.041, 0.021				
*WPPP* × *z_MOP*	−0.050 ***	−2.669	0.008	−0.086, −0.013				
*LNWPPPI*					−0.000	−0.108	0.914	−0.006, 0.006
*z_LNWPPPI* × *z_MOP*					−0.009 *	−1.890	0.059	−0.018, 0.000
Obs.	3204				3204			
R-sq	0.446				0.443			
Model	FE				FE			
Control	YES				YES			
City_FE	YES				YES			
Year_FE	YES				YES			
Cities	267				267			

Note: To facilitate interpretation of the meaning of the result coefficients, the variables in the interaction term are centralized and distinguished by the prefix “*z_*”; *** *p* < 0.01, * *p* < 0.10.

**Table 7:** The impact of fiscal decentralization of cities.

	(1)				(2)			
	*LNSW*				*LNSW*			
	Coef.	t	p	95% CI	Coef.	t	p	95% CI
Panel A								
*WPPP*	−0.032 **	−2.392	0.017	−0.057, −0.006				
*WPPP* × *z_CZFQ*	−0.022	−0.443	0.658	−0.120, 0.076				
*LNWPPPI*					−0.002	−0.798	0.425	−0.007, 0.003
*LNWPPPI* × *z_CZFQ*					−0.003	−0.285	0.776	−0.022, 0.017
Obs.	3024				3024			
R-sq	0.426				0.425			
Model	FE				FE			
Control	YES				YES			
City_FE	YES				YES			
Year_FE	YES				YES			
Cities	252				252			
Panel B								
*WPPP*	−0.045 ***	−2.617	0.009	−0.079, −0.011				
*WPPP* × *CS2*	0.009	0.511	0.610	−0.027, 0.046				
*WPPP* × *CS3*	0.031	1.590	0.112	−0.007, 0.069				
*LNWPPPI*					−0.004	−1.179	0.238	−0.010, 0.003
*LNWPPPI* × *CS2*					0.001	0.278	0.781	−0.006, 0.008
*LNWPPPI* × *CS3*					0.004	1.174	0.240	−0.003, 0.012
Obs.	3024				3024			
R-sq	0.426				0.425			
Model	FE				FE			
Control	YES				YES			
City_FE	YES				YES			
Year_FE	YES				YES			
Cities	252				252			

Note: To facilitate interpretation of the meaning of the result coefficients, the variables in the interaction term are centralized and distinguished by the prefix “*z_*”; *** *p* < 0.01, ** *p* < 0.05.

**Table 8:** The impact of national demonstration PPPSTs.

	(1)				(2)			
	*LNSW*				*LNSW*			
	Coef.	t	p	95% CI	Coef.	t	p	95% CI
*WPPP*	−0.010	−0.571	0.568	−0.045, 0.025				
*WPPP* × *NATION*	−0.034 **	−2.045	0.041	−0.066, −0.001				
*LNWPPPI*					0.002	0.596	0.551	−0.005, 0.010
*z_LNWPPPI* × *NATION*					−0.008 **	−2.020	0.043	−0.015, −0.000
Obs.	3204				3204			
R-sq	0.445				0.444			
Model	FE				FE			
Control	YES				YES			
City_FE	YES				YES			
Year_FE	YES				YES			
Cities	267				267			

Note: To facilitate interpretation of the meaning of the result coefficients, the variables in the interaction term are centralized and distinguished by the prefix “z_”; ** *p* < 0.05.

## Data Availability

Not applicable.
